# *N*-(4-Isocyano­phen­yl)succinamic acid

**DOI:** 10.1107/S1600536812025226

**Published:** 2012-06-13

**Authors:** Lauren E. Burnham, Katrina J. Gano, Amber M. Young, John M. Risley, Daniel S. Jones

**Affiliations:** a Department of Chemistry, The University of North Carolina at Charlotte, 9201 University City Blvd., Charlotte, NC 28223, USA

## Abstract

In the crystal structure of the title compound, C_11_H_10_N_2_O_3_, inversion-related mol­ecules are connected by pairs of O—H⋯O hydrogen bonds. With the exception of the atoms in the carb­oxy­lic acid group, the non-H atoms are roughly coplanar with a maximum deviation from the mean plane of 0.270 (1) Å for the C atom to which the carb­oxy­lic group is attached. The C atom of the carb­oxy­lic group lies 1.730 (2) Å from the mean plane.

## Related literature

For the structure of 4-isocyano­aniline see: Britton (1993 ▶). For details of the enzyme-catalysed reaction, see: Risley *et al.* (2001 ▶); Du & Risley (2003 ▶). For the synthetic procedures, see: Heinze & Jacob (2003 ▶); Kar & Argade (2002 ▶).
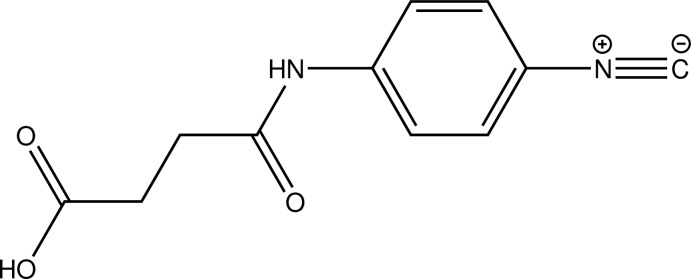


## Experimental

### 

#### Crystal data

C_11_H_10_N_2_O_3_
*M* *_r_* = 218.21
Monoclinic, 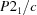

*a* = 5.0974 (2) Å
*b* = 16.2774 (7) Å
*c* = 12.5674 (6) Å
β = 94.771 (4)°
*V* = 1039.13 (8) Å^3^
*Z* = 4
Cu *K*α radiation
μ = 0.87 mm^−1^
*T* = 100 K
0.42 × 0.09 × 0.08 mm

#### Data collection

Agilent Xcalibur Atlas Gemini ultra diffractometer
Absorption correction: multi-scan (*CrysAlis PRO*; Agilent, 2011 ▶) *T*_min_ = 0.746, *T*_max_ = 1
7543 measured reflections
1839 independent reflections
1527 reflections with *I* > 2σ(*I*)
*R*_int_ = 0.035

#### Refinement

*R*[*F* ^2^ > 2σ(*F* ^2^)] = 0.038
*wR*(*F* ^2^) = 0.096
*S* = 1.07
1839 reflections
153 parameters
H atoms treated by a mixture of independent and constrained refinement
Δρ_max_ = 0.21 e Å^−3^
Δρ_min_ = −0.25 e Å^−3^

### 

Data collection: *CrysAlis PRO* (Agilent, 2011 ▶); cell refinement: *CrysAlis PRO*; data reduction: *CrysAlis PRO*; program(s) used to solve structure: *SHELXS97* (Sheldrick, 2008 ▶); program(s) used to refine structure: *SHELXL97* (Sheldrick, 2008 ▶); molecular graphics: *ORTEP-3 for Windows* (Farrugia, 1997 ▶) and *Mercury* (Macrae *et al.*, 2006 ▶); software used to prepare material for publication: *WinGX* publication routines (Farrugia, 1999 ▶).

## Supplementary Material

Crystal structure: contains datablock(s) global, I. DOI: 10.1107/S1600536812025226/fj2512sup1.cif

Structure factors: contains datablock(s) I. DOI: 10.1107/S1600536812025226/fj2512Isup2.hkl

Supplementary material file. DOI: 10.1107/S1600536812025226/fj2512Isup3.cml

Additional supplementary materials:  crystallographic information; 3D view; checkCIF report

## Figures and Tables

**Table 1:** Hydrogen-bond geometry (Å, °)

*D*—H⋯*A*	*D*—H	H⋯*A*	*D*⋯*A*	*D*—H⋯*A*
O3—HO3⋯O2^i^	0.94 (3)	1.72 (3)	2.6646 (17)	177 (2)

Symmetry code: (i) 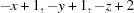.

**Fig. 1.**
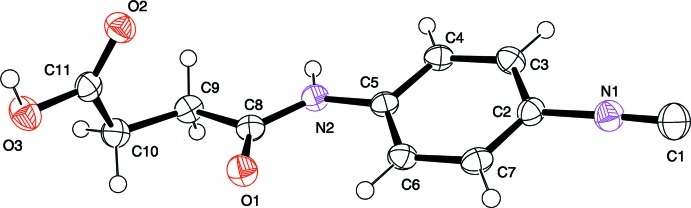
View of the title compound. Displacement ellipsoids are drawn at the 50% probability level.

**Fig. 2.**
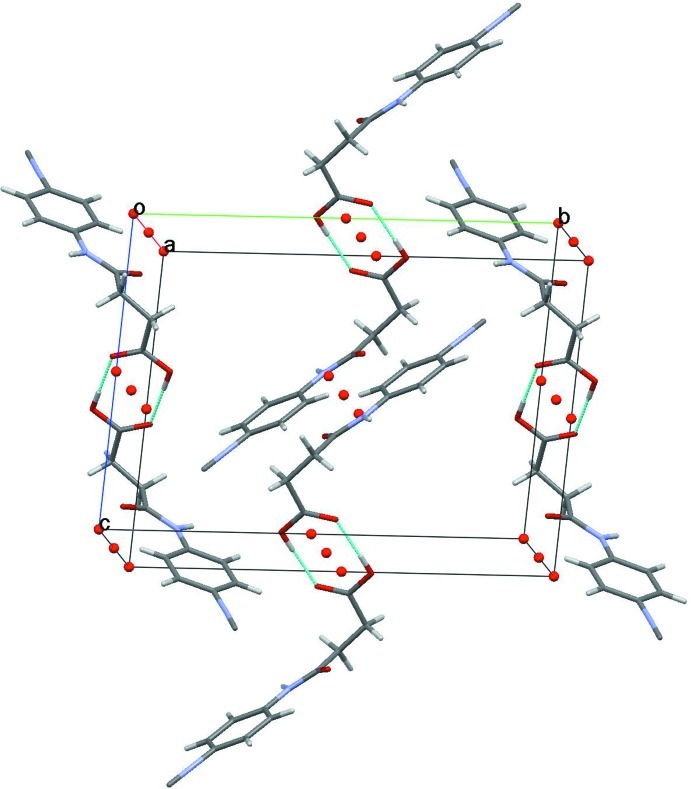
Crystal packing diagram of the title compound showing hydrogen bonding.

## supplementary crystallographic information

### Comment

Glycosylasparaginase is a key lysosomal enzyme in the catabolism of N-linked glycoproteins. The natural substrate for the enzyme is *N*^4^-(2-acetamido-2-deoxy-β-*D*-glucopyranosyl)-L-asparagine. In a study of the enzyme-catalyzed reaction in which the structure of the amino acid part of the natural substrate was altered (and the sugar was unchanged), it was found that the binding sites on the enzyme for the sugar and the α-carboxyl group were sufficient for the enzyme to act on the substrate, with the α-amino group acting as an "anchor" in the binding site for the substrate (Risley *et al.*, 2001). In a follow-up study, a series of *N*^4^-(4'-substituted phenyl)-L-asparagines were synthesized in which the structure of the amino acid part of the natural substrate was unchanged, but the sugar was substituted with *para*-substituted anilines. All of these β-anilides of asparagine were substrates for the enzyme (Du & Risley, 2003).

In order to better understand the role of the α-amino group in the binding to the enzyme, results from the two studies were used to design a series of *N*^4^-(4'-substituted phenyl)succinamic acids in which the structure of the amino acid part of the natural substrate was changed to succinamic acid (the α-amino group was replaced with a hydrogen) and the sugar part was substituted with *para*-substituted anilines. One of the substrate analogues synthesized was the title compound, with the atypical electron-withdrawing isocyano group that should make 4-isocyanoaniline a very good leaving group in the hydrolysis of the amide bond during the enzyme-catalyzed reaction. Initial studies of these analogues with the enzyme, however, indicated that they were neither substrates nor inhibitors. These initial studies would indicate that, for anilide substrates, the α-amino group is required for binding of the substrate to the active site of the enzyme, in contrast to the sugar substrates for which the α-amino group was not required.

The pair of molecules related by the inversion center at (1/2,1/2,0) are joined by two symmetry-equivalent O—H···O hydrogen bonds, as shown in Figure 2 and described in Table 1. The non-hydrogen atoms of the molecule are nearly planar, with the exception of those in the carboxylic acid group. A mean plane was fit to all of the non-hydrogen atoms except those of the carboxylic group; the maximum deviation of the fitted atoms is 0.270 (1) Å, for C10. The carbon of the carboxylic group, C11, lies 1.730 (2) Å from the mean plane.

### Experimental

4-Isocyanoaniline was synthesized as described in the literature (Heinze & Jacob, 2003). The general procedure described by Kar and Argade (Kar & Argade, 2002) was then used to synthesize the title compound. 4-Isocyanoaniline (1.18 g, 0.01 mol) and succinic anhydride (1 g, 0.01 mol) were added to benzene:1,4-dioxane (2:1, 60 ml) and stirred at room temperature for 24 h to give needles that were collected and washed with the benzene:dioxane solvent. Yield 1.22 g (0.006 mol, 56%).

Mp 424 K. ^1^H NMR: δ_H_ (300.53 MHz, CD_3_OD) 2.677 (4*H*, center of AA'BB' spectrum: Δn_AB_ 4.10 Hz, *J*_AB_ 7.44 Hz, *J*_AB'_ 6.00 Hz, *J*_AA'_ -16.00 Hz, *J*_BB'_ -17.00 Hz, H-2,3), 7.378 (2*H*, BB' of AA'BB', *J*_AB_ 8.56, *J*_AB'_ 0.40, *J*_BB'_ 2.50, H-3',5'), 7.655 (2*H*, AA' of AA'BB', *J*_AA'_ 2.20, H-2',6'), 9.510 (1*H*, s, NH), OH not observed. ^13^C NMR: δ_C_ (125.77 MHz, DMSO-*d*_6_) 28.634 (C2), 31.122 (C3), 119.287 (C3'/5'), 119.358 (C5'/3'), 120.064 (C4'), 126.991 (C2'/6'), 127.161 (C6'/2'), 140.404 (C1'), 162.982 (NC), 170.672 (C4), 173.784 (C1).

### Refinement

The hydrogen atoms on N2 (HN2) and O3 (HO3) were refined isotropically. The remaining H atoms were included in calculated positions and treated as riding atoms. Aromatic C—H bond lengths were 0.93 Å and methylene C—H bond lengths were 0.97 Å, with *U*_iso_(H) = 1.2 *U*_eq_(C) in both cases.

### Crystal data

**Table:**

C_11_H_10_N_2_O_3_	*F*(000) = 456
*M**_r_* = 218.21	*D*_x_ = 1.395 Mg m^−^^3^
Monoclinic, *P*2_1_/*c*	Cu *K*α radiation, λ = 1.54182 Å
Hall symbol: -P 2ybc	Cell parameters from 2647 reflections
*a* = 5.0974 (2) Å	θ = 3.5–66.9°
*b* = 16.2774 (7) Å	µ = 0.87 mm^−^^1^
*c* = 12.5674 (6) Å	*T* = 100 K
β = 94.771 (4)°	Prism, colourless
*V* = 1039.13 (8) Å^3^	0.42 × 0.09 × 0.08 mm
*Z* = 4	

### Data collection

**Table:**

Agilent Xcalibur Atlas Gemini ultra diffractometer	1839 independent reflections
Graphite monochromator	1527 reflections with *I* > 2σ(*I*)
Detector resolution: 10.4419 pixels mm^-1^	*R*_int_ = 0.035
ω scans	θ_max_ = 67.0°, θ_min_ = 4.5°
Absorption correction: multi-scan (*CrysAlis PRO*; Agilent, 2011)	*h* = −5→6
*T*_min_ = 0.746, *T*_max_ = 1	*k* = −19→19
7543 measured reflections	*l* = −14→14

### Refinement

**Table:**

Refinement on *F*^2^	Primary atom site location: structure-invariant direct methods
Least-squares matrix: full	Secondary atom site location: difference Fourier map
*R*[*F*^2^ > 2σ(*F*^2^)] = 0.038	Hydrogen site location: inferred from neighbouring sites
*wR*(*F*^2^) = 0.096	H atoms treated by a mixture of independent and constrained refinement
*S* = 1.07	*w* = 1/[σ^2^(*F*_o_^2^) + (0.0476*P*)^2^ + 0.257*P*] where *P* = (*F*_o_^2^ + 2*F*_c_^2^)/3
1839 reflections	(Δ/σ)_max_ < 0.001
153 parameters	Δρ_max_ = 0.21 e Å^−^^3^
0 restraints	Δρ_min_ = −0.25 e Å^−^^3^

### Special details

**Table:**

Geometry. All s.u.'s (except the s.u. in the dihedral angle between two l.s. planes) are estimated using the full covariance matrix. The cell s.u.'s are taken into account individually in the estimation of s.u.'s in distances, angles and torsion angles; correlations between s.u.'s in cell parameters are only used when they are defined by crystal symmetry. An approximate (isotropic) treatment of cell s.u.'s is used for estimating s.u.'s involving l.s. planes.
Refinement. Refinement of *F*^2^ against ALL reflections. The weighted *R*-factor *wR* and goodness of fit *S* are based on *F*^2^, conventional *R*-factors *R* are based on *F*, with *F* set to zero for negative *F*^2^. The threshold expression of *F*^2^ > 2σ(*F*^2^) is used only for calculating *R*-factors(gt) *etc*. and is not relevant to the choice of reflections for refinement. *R*-factors based on *F*^2^ are statistically about twice as large as those based on *F*, and *R*- factors based on ALL data will be even larger.

### Fractional atomic coordinates and isotropic or equivalent isotropic displacement parameters (Å^2^)

**Table:**

	*x*	*y*	*z*	*U*_iso_*/*U*_eq_	
HN2	−0.184 (4)	0.6215 (11)	0.6545 (15)	0.028 (5)*	
HO3	0.484 (5)	0.4256 (16)	0.9999 (19)	0.064 (7)*	
O3	0.3472 (2)	0.40108 (7)	0.95600 (10)	0.0327 (3)	
O2	0.2537 (2)	0.53307 (7)	0.92254 (10)	0.0307 (3)	
O1	0.2238 (2)	0.48805 (7)	0.66450 (9)	0.0302 (3)	
N2	−0.0524 (3)	0.59719 (8)	0.63180 (11)	0.0246 (3)	
N1	0.4162 (3)	0.77295 (8)	0.31697 (11)	0.0286 (3)	
C4	−0.0207 (3)	0.71789 (10)	0.52404 (13)	0.0254 (4)	
H4	−0.1583	0.7404	0.5585	0.03*	
C7	0.3901 (3)	0.65037 (10)	0.42199 (13)	0.0261 (4)	
H7	0.5285	0.6283	0.3875	0.031*	
C2	0.2966 (3)	0.72819 (10)	0.39560 (12)	0.0248 (4)	
C6	0.2774 (3)	0.60562 (10)	0.49959 (13)	0.0254 (4)	
H6	0.339	0.5531	0.517	0.03*	
C3	0.0902 (3)	0.76253 (10)	0.44606 (13)	0.0268 (4)	
H3	0.028	0.8147	0.4275	0.032*	
C8	0.0281 (3)	0.52704 (10)	0.68370 (13)	0.0246 (4)	
C9	−0.1502 (3)	0.49859 (10)	0.76713 (13)	0.0269 (4)	
H9A	−0.3155	0.4793	0.7321	0.032*	
H9B	−0.188	0.5445	0.8127	0.032*	
C5	0.0710 (3)	0.63904 (9)	0.55200 (12)	0.0234 (3)	
C11	0.2059 (3)	0.46016 (10)	0.90780 (13)	0.0256 (4)	
C1	0.5262 (4)	0.80691 (11)	0.25313 (15)	0.0351 (4)	
C10	−0.0224 (3)	0.43002 (10)	0.83482 (14)	0.0281 (4)	
H10C	−0.1525	0.4056	0.8772	0.034*	
H10D	0.0385	0.3877	0.7884	0.034*	

### Atomic displacement parameters (Å^2^)

**Table:**

	*U*^11^	*U*^22^	*U*^33^	*U*^12^	*U*^13^	*U*^23^
O3	0.0293 (7)	0.0276 (6)	0.0401 (7)	−0.0031 (5)	−0.0031 (5)	0.0038 (5)
O2	0.0316 (6)	0.0264 (6)	0.0338 (7)	−0.0021 (5)	0.0002 (5)	0.0004 (5)
O1	0.0254 (6)	0.0309 (6)	0.0350 (7)	0.0056 (5)	0.0068 (5)	0.0026 (5)
N2	0.0213 (7)	0.0262 (7)	0.0271 (7)	0.0038 (6)	0.0074 (6)	−0.0001 (6)
N1	0.0269 (7)	0.0296 (7)	0.0297 (8)	−0.0010 (6)	0.0039 (6)	0.0007 (6)
C4	0.0215 (8)	0.0264 (8)	0.0287 (9)	0.0027 (6)	0.0050 (7)	−0.0037 (6)
C7	0.0222 (8)	0.0299 (8)	0.0268 (9)	0.0000 (6)	0.0049 (7)	−0.0059 (7)
C2	0.0238 (8)	0.0273 (8)	0.0235 (8)	−0.0026 (6)	0.0038 (7)	−0.0006 (6)
C6	0.0255 (8)	0.0234 (8)	0.0277 (8)	0.0028 (6)	0.0042 (7)	−0.0025 (6)
C3	0.0248 (8)	0.0234 (8)	0.0323 (9)	0.0012 (6)	0.0032 (7)	−0.0002 (7)
C8	0.0213 (8)	0.0263 (8)	0.0259 (8)	−0.0012 (6)	−0.0002 (6)	−0.0029 (6)
C9	0.0226 (8)	0.0309 (9)	0.0275 (9)	−0.0015 (7)	0.0035 (7)	−0.0018 (7)
C5	0.0219 (8)	0.0247 (8)	0.0234 (8)	−0.0010 (6)	0.0013 (6)	−0.0031 (6)
C11	0.0252 (8)	0.0278 (9)	0.0248 (9)	−0.0008 (7)	0.0083 (7)	0.0029 (7)
C1	0.0313 (9)	0.0378 (10)	0.0365 (10)	−0.0023 (8)	0.0054 (8)	0.0037 (8)
C10	0.0266 (9)	0.0282 (8)	0.0302 (9)	−0.0035 (7)	0.0066 (7)	0.0007 (7)

### Geometric parameters (Å, º)

**Table:**

O3—C11	1.318 (2)	C7—C2	1.384 (2)
O3—HO3	0.94 (3)	C7—H7	0.93
O2—C11	1.2226 (19)	C2—C3	1.390 (2)
O1—C8	1.2235 (19)	C6—C5	1.397 (2)
N2—C8	1.361 (2)	C6—H6	0.93
N2—C5	1.404 (2)	C3—H3	0.93
N2—HN2	0.85 (2)	C8—C9	1.516 (2)
N1—C1	1.157 (2)	C9—C10	1.517 (2)
N1—C2	1.407 (2)	C9—H9A	0.97
C4—C3	1.378 (2)	C9—H9B	0.97
C4—C5	1.401 (2)	C11—C10	1.503 (2)
C4—H4	0.93	C10—H10C	0.97
C7—C6	1.380 (2)	C10—H10D	0.97
			
C11—O3—HO3	108.1 (15)	O1—C8—C9	121.58 (15)
C8—N2—C5	127.90 (14)	N2—C8—C9	114.42 (14)
C8—N2—HN2	116.4 (12)	C8—C9—C10	111.00 (13)
C5—N2—HN2	115.4 (12)	C8—C9—H9A	109.4
C1—N1—C2	176.27 (17)	C10—C9—H9A	109.4
C3—C4—C5	120.89 (14)	C8—C9—H9B	109.4
C3—C4—H4	119.6	C10—C9—H9B	109.4
C5—C4—H4	119.6	H9A—C9—H9B	108
C6—C7—C2	119.82 (14)	C6—C5—C4	119.16 (14)
C6—C7—H7	120.1	C6—C5—N2	123.20 (14)
C2—C7—H7	120.1	C4—C5—N2	117.64 (13)
C7—C2—C3	121.15 (15)	O2—C11—O3	122.96 (15)
C7—C2—N1	118.79 (14)	O2—C11—C10	122.95 (15)
C3—C2—N1	120.06 (14)	O3—C11—C10	114.08 (14)
C7—C6—C5	120.10 (15)	C11—C10—C9	112.13 (13)
C7—C6—H6	119.9	C11—C10—H10C	109.2
C5—C6—H6	119.9	C9—C10—H10C	109.2
C4—C3—C2	118.89 (15)	C11—C10—H10D	109.2
C4—C3—H3	120.6	C9—C10—H10D	109.2
C2—C3—H3	120.6	H10C—C10—H10D	107.9
O1—C8—N2	123.98 (15)		

### Hydrogen-bond geometry (Å, º)

**Table:**

*D*—H···*A*	*D*—H	H···*A*	*D*···*A*	*D*—H···*A*
O3—H*O*3···O2^i^	0.94 (3)	1.72 (3)	2.6646 (17)	177 (2)

Symmetry code: (i) −*x*+1, −*y*+1, −*z*+2.
